# Lithium Toxicity in the Setting of Nonsteroidal Anti-Inflammatory Medications

**DOI:** 10.1155/2013/839796

**Published:** 2013-04-03

**Authors:** Syed Hassan, Fatima Khalid, Zaid Alirhayim, Syed Amer

**Affiliations:** ^1^Department of Internal Medicine, Henry Ford Health System, 2799 West Boulevard, Detroit, MI 48202, USA; ^2^Department of Internal Medicine, Brookdale Hospital and Medical Center, Detroit, MI, USA

## Abstract

Lithium toxicity is known to affect multiple organ systems, including the central nervous system. Lithium levels have been used in the diagnosis of toxicity and in assessing response to management. There is evidence that nonsteroidal anti-inflammatory medications (NSAIDs) can increase lithium levels and decrease renal lithium clearance. We present a case of lithium toxicity, which demonstrates this effect and also highlights the fact that lithium levels do not correlate with clinical improvement, especially the neurological deficit.

## 1. Introduction 

Lithium is used as a first line maintenance therapy for bipolar disorder and as a mood stabilizer [1–3]. However the therapeutic window is very narrow, and it has a broader side effect profile making it difficult for a clinician to manage it and needs constant serial blood lithium concentration monitoring [4]. Lithium toxicity is more pronounced in patients with decreased renal function and reduced volume of distribution [5, 6]. Also nephrotoxic medications such as COX-2 inhibitors and NSAID can affect the pharmacokinetics and can lead to serious adverse effects [7]. 

## 2. Case

This is a 51-year-old African American male with a history of schizophrenia and bipolar disorder diagnosed at the age of 26. He lives in an extended care facility and is seen by his psychiatry every six months. His list of medications includes lithium, valproate, quetiapine, and risperidone. He presented to the emergency department with confusion, alert and oriented to his name, dysarthria, abnormal gait, and diarrhea. He was accompanied by his caregiver who stated that his symptoms started four days ago and is progressively getting worse. The only pertinent history the caregiver provided was that all these symptoms started 2 days after his visit to a dentist for removal of an infected molar. At that time he was prescribed ibuprofen 800 mg three times a day for 5 days. 

In the emergency department, his laboratory values were significant for elevated lithium level (3 mmol/L) with mild renal failure (serum creatinine 1.6 mg/dL) secondary to dehydration. He was initially treated with intravenous hydration supportive care was provided, and, he was then transferred to the inpatient service. Hemodialysis was not initiated as renal function along with his lithium levels improved rapidly within 24 hours. However, over the next few days his serum lithium levels normalized (1 mmol/L) without improvement in his mental status. Subsequently, he required intubation and was transferred to the intensive care unit. His infectious/metabolic workup was negative; an unremarkable computed tomography scan of the head and an electroencephalography revealed metabolic encephalopathy. With the next few days of supportive care, the patient was extubated, and his mental status returned to baseline. He was subsequently discharged from the hospital and sent back to his facility and is currently followed by his primary care physician.

## 3. Discussion

We report a case of lithium toxicity in the setting of NSAID use, where a patient had normalized serum lithium levels with delayed improvement in mental status. It is recommended that lithium levels should be checked every 4-5 days after starting an NSAID to assess for toxicity. We emphasize that lithium levels may be helpful in the primary diagnosis of toxicity, and it loosely correlates with serum drug concentration. Thus management of toxicity should be dictated by clinical signs and symptoms but not serum concentration. In cases of acute toxicity, lithium is mainly an extracellular water soluble ion rapidly cleared by intravenous hydration or hemodialysis [4, 8]. However, in cases of toxicity following chronic lithium ingestion, intracellular and intracerebral concentrations are high. When the serum lithium level normalizes, intracellular concentrations remain elevated, and further clinical decompensation is possible. This occurs because lithium equilibrates slowly between both compartments, requiring multiple prolonged hemodialysis treatment sessions [9]. Reviewing this case retrospectively reinforces the current guidelines for the management of lithium toxicity. Any patient who comes in with altered mental status associated with toxic lithium levels (≥2.5 mmol/L) should undergo multiple prolonged hemodialysis sessions to adequately deplete intracellular lithium. Also clinicians should be aware of the fact of lithium toxicity induced by NSAIDS which is the commonly available over the counter medication.
